# 

**Published:** 1897-09

**Authors:** 


### CONTENTS.

COMMUNICATIONS.

President's Address.......... 417
Treatment of Abscess.......... 421
Point of Contact.......... 425
To What Extent and When are We Justified in Using Cataphoresis? Is There Danger of Injuring the Dental Pulp or other Tissues, by its use.......... 432
Dental Bacteriology.......... 441

PROCEEDINGS.

National Association of Dental Faculties.......... 451
Annual Address, 1897.......... 458

SELECTIONS.

Medical Superstitions.......... 461
Pain and the Surgeon.......... 462
Kaiserling's Method for the Preservation of Specimens with their Natural Colors.......... 463

COMMENCEMENTS.

Meharry Dental Commencement.......... 463

EDITORIAL

The Meetings at Old Point Comfort, 1897.......... 464
New College at Indianapolis.......... 466
The American Dental Weekly.......... 467
A New Work on Surgery.......... 467
Ohio State Dental Society.......... 467
Bibliographical.......... 468

